# The in vitro and in vivo anti-virulent effect of organic acid mixtures against *Eimeria tenella* and *Eimeria bovis*

**DOI:** 10.1038/s41598-021-95459-9

**Published:** 2021-08-10

**Authors:** Igori Balta, Adela Marcu, Mark Linton, Carmel Kelly, Lavinia Stef, Ioan Pet, Patrick Ward, Gratiela Gradisteanu Pircalabioru, Carmen Chifiriuc, Ozan Gundogdu, Todd Callaway, Nicolae Corcionivoschi

**Affiliations:** 1 Bacteriology Branch, Veterinary Sciences Division, Agri-Food and Biosciences Institute, 18a Newforge Lane, Belfast, BT9 5PX Northern Ireland UK; 2 Faculty of Animal Science and Biotechnologies, University of Agricultural Sciences and Veterinary Medicine, 400372 Cluj-Napoca, Romania; 3 Faculty of Bioengineering of Animal Resources, Banat University of Agricultural Sciences and Veterinary Medicine-King Michael I of Romania, Timisoara, Romania; 4 Auranta, Nova UCD, Belfield, Dublin 4, Ireland; 5 Research Institute of University of Bucharest, 300645 Bucharest, Romania; 6 Department of Infection Biology, Faculty of Infectious and Tropical Diseases, London School of Hygiene and Tropical Medicine, London, UK; 7 Department of Animal and Dairy Science, University of Georgia, Athens, GA USA

**Keywords:** Parasite host response, Parasite immune evasion

## Abstract

*Eimeria tenella* and *Eimeria bovis* are complex parasites responsible for the condition of coccidiosis, that invade the animal gastrointestinal intestinal mucosa causing severe diarrhoea, loss of appetite or abortions, with devastating impacts on the farming industry. The negative impacts of these parasitic infections are enhanced by their role in promoting the colonisation of the gut by common foodborne pathogens. The aim of this study was to test the anti-Eimeria efficacy of maltodextrin, sodium chloride, citric acid, sodium citrate, silica, malic acid, citrus extract, and olive extract individually, in vitro and in combination, in vivo. Firstly, in vitro infection models demonstrated that antimicrobials reduced (p < 0.05), both singly and in combination (AG), the ability of *E. tenella* and *E. bovis* to infect MDBK and CLEC-213 epithelial cells, and the virulence reduction was similar to that of the anti-coccidial drug Robenidine. Secondly, using an in vivo broiler infection model, we demonstrated that AG reduced (p = 0.001) *E. tenella* levels in the caeca and excreted faeces, reduced inflammatory oxidative stress, improved the immune response through reduced ROS, increased Mn-SOD and SCFA levels. Levels of IgA and IgM were significantly increased in caecal tissues of broilers that received 0.5% AG and were associated with improved (p < 0.0001) tissue lesion scores. A prophylactic approach increased the anti-parasitic effect in vivo, and results indicated that administration from day 0, 5 and 10 post-hatch reduced tissue lesion scores (p < 0.0001) and parasite excretion levels (p = 0.002). Conclusively, our in vitro and in vivo results demonstrate that the natural antimicrobial mixture (AG) reduced parasitic infections through mechanisms that reduced pathogen virulence and attenuated host inflammatory events.

## Introduction

Unicellular protozoa of the phylum Apicomplexa, such as *Eimeria* spp., cause severe infections in livestock (coccidiosis), particularly in poultry and cattle^1^. At a global scale, avian coccidiosis alone is responsible for more than $3 billion in economic losses to the poultry industry^2^. While other *Eimeria* spp., cause disease significant consideration of *E. bovis* is warranted because of its association with severe typhlocolitis in calves^3^. At host level *Eimeria* inhibits the activation of NF-κB, impairs gene expression of immunomodulatory molecules, modulates cell apoptosis and cholesterol metabolism, and reduces the integrity of the cellular cytoskeleton^4^. These effects are manifested through acute haemorrhagic diarrhoea, body dehydration, weight loss and drastic decreases in growth dynamics. While most coccidial infections are asymptomatic, clinical signs occur especially in immunocompromised animals^5^. However, even in the absence of symptomatic infection, gastrointestinal function and composition of the resident microbiota are altered by these protozoa, leading to further mucosal injuries. Moreover, these coccidial mucosal injuries are also a predisposing factor for further bacterial caused necrotic enteritis (e.g. *Salmonella enterica* Typhimurium and *Clostridium perfringens*)^6,7^.

The infectivity potential of oocysts is dependent upon the sporulation rate and wall structure, which for *Eimeria* spp. provides a strong defence and resilience to chemical, mechanical and physically damaging stressors including anti-coccidia as well as other antimicrobial substances^8^. Evidence of drug resistance in *Eimeria* spp., suggests urgency to develop novel alternative approaches which can optimise and enhance the efficacy of existing control strategies. Modern biotechnology offers promising natural alternatives to anti-parasitic, anticoccidial and antibiotic drugs. Recent developments in this area paved the way for new, more effective and environmentally friendly alternatives to tackle coccidial infections. These developments have placed host safety as the top priority, ensuring that few if any secondary effects are expressed as are often seen for antibiotics, anticoccidial or anti-parasitic drugs^9,10^.

Phytobiotics and organic acids often inhibit pathogens, including parasites, and can modulate animal gastrointestinal tract health via multiple mechanisms^5,7,9,11^. Phytochemicals, such as saponins, exhibit a unique ability to adhere to the protozoan cholesterol cellular membrane of *Eimeria* spp., causing further lysis and cellular death^11,12^. Moreover, saponins used with thymol and carvacrol synergistically inhibited invasion of MDBK cells by *Eimeria* sporozoites at concentrations of 3.5–5 ppm^12^. Saponins extracted from *Yucca schidigera* mitigated inflammatory responses in *Eimeria*-infected broilers^10^. Further, low concentrations of phytochemicals from *Biden pilosa* (cytopiloyne) suppressed *E. tenella* and excretion of oocysts, reducing severity of clinical symptoms via the promotion of T cell-mediated immunity^13^. The direct anti-coccidial mechanism was associated with inhibition of oocyst sporulation, attenuation of sporozoite invasiveness, and interference with *Eimeria* schizonts development^13^.

Natural antimicrobial phytochemical blends enriched with organic acids are promising additions to pathogen control programmes because they can be easily integrated in animal feeds^9^. Commercial blends of organic acids such as Acidomix (Ammonium formate, formic acid, Ammonium Propionate), Activate (2-hydroxyl-4-calcium butyrate, fumaric acid, Benzoic acid) and Lacplus (lactic acid, citric acid, fumaric acid, phosphoric acid) reduced lesion scores, oocyst indices, increased body weight, and improved feed conversion ratio in *E. tenella*-infected chicks^1^*.* Moreover, the treatment boosted local gene expression of important cytokines and chemokines (e.g., IL-8, IL-15, IL-17 and IFN-γ) in the spleen and cecum^2^. A blend of fatty acids, organic acids, and phytochemicals upregulated jejunal gene expression of the parasitic infection indicator IFN-Ɣ, and epithelial permeability regulator claudin-1 in broilers^7^. Currently, little is known about the efficacy of phytochemicals and/or their metabolites, prebiotics or organic acids against bovine coccidiosis^14^.

The in vitro effect of natural antimicrobials including maltodextrin^15^, citric acid^16^, sodium citrate^17^, silica^18^, malic acid^19^, citrus extract^20^, and olive extract^21^ against parasitic infections have been previously described. However, most have a limited impact when administered individually. We have shown previously that when used in combination the anti-bacterial effect^22^ was enhanced both in vitro and in vivo. The present study investigates the anti-parasitic effect in vitro, based on their individually anti-parasitic potency against *E. tenella* and *E. bovis*. Following our in vitro results, we have also examined the impact of mixed natural antimicrobials on *E. tenella* infections in chicken broilers using a mixture of citric acid, sodium citrate, silica, malic acid, citrus extract, and olive extract.

## Results

### Cytotoxicity, in vitro inhibition of virulence and of sporozoite sporulation

**Figure 1:**
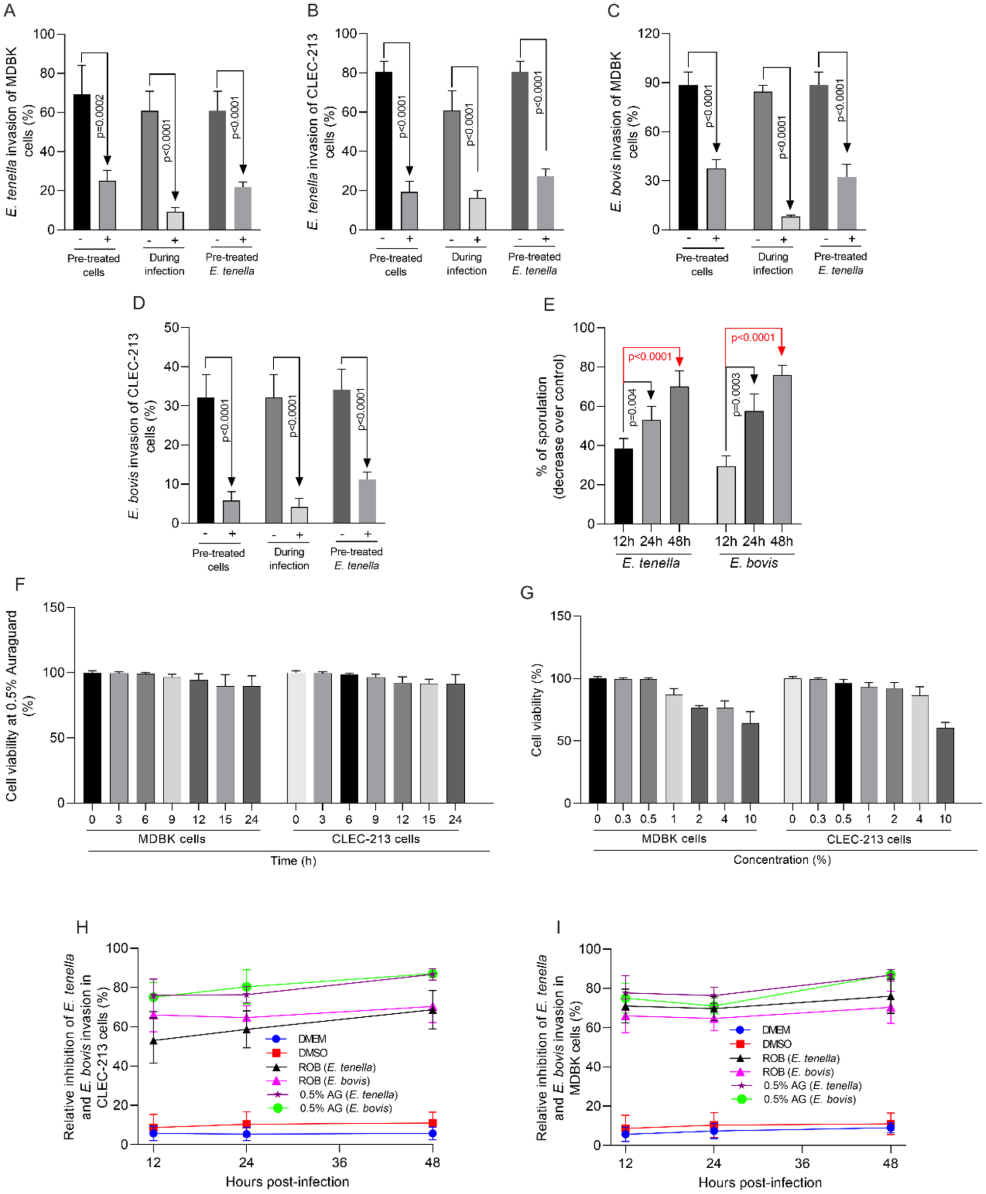
Effect of a mixture of natural antimicrobials (AG) on in vitro cell invasiveness by *E. tenella* and *E. bovis* using MDBK, and CLEC cells. (**A**) The effect of AG on the percentual invasion efficiency for *E. tenella* in MDBK cells and (**B**) for invasion of CLEC-213; (**C**) describes the effect of AG on the invasion of *E. bovis* in MDBK cells and (**D**) in CLEC-213 cells; (**E**) the in vitro effect of the antimicrobial mixture (AG) on *E. tenella* and *E. bovis* oocyst sporulation. (**F**) Describes the in vitro effect of 0.5% AG on cell viability over 24 h and in (**G**) cells were treated with a series of concentrations of AG (0; 0.3; 0.5; 1; 2; 4 and 10%) for 24 h estimated by the MTT assay and expressed as a percentage based on the untreated control cells. Values are the mean ± SE (*n* = 6). (**H**) The impact of the antimicrobial mixture (AG) (0.5%) on sporozoite counts by comparison with the anticoccidial robenidine (ROB) (5 μg/ml) in CLEC-213 and Panel I in MDBK cells. The experiments were done in triplicate and in three separate occasions.

Firstly, screening of the individual components shows that the number of oocysts was decreased by treatment in a dose dependent fashion at concentrations between 0.05 and 0.96 mg/ml and was expressed as lethal concentration 50% (LC_50_) (Table 1). Using their individual anti-parasitic effects we have then examined the effects of the concentration (0–10%) of the antimicrobials, in mixture, on numbers of oocysts. Lethal concentration 50% (LC_50_) shows that at 0.5% the number of oocysts were reduced by approximately 50% (Table 2), *E. tenella* LC_50_ = 47.9% and *E. bovis* LC_50_ = 50.2%. Effects of AG on all cell lines viability found that 0.5% AG did not inhibit the proliferation of MDBK and CLEC-213 and maintained a 98.9% viability (Fig. 1). Addition of 0.5% AG was used to estimate the impact of AG on epithelial cell invasion in vitro (Fig. 1F,G).

**Table 1:** Lethal concentration 50 (LC_50_) at which the components of the antimicrobial mixture reduced number of oocysts to half of the initial numbers.

Antimicrobial	LC_50_ (mg/ml)	
	*E. tenella*	*E. bovis*
Maltodextrin	0.25 ± 0.04	0.29 ± 0.26
Sodium chloride	0.05 ± 0.19	0.07 ± 0.07
Citric acid	2.06 ± 0.15	2.21 ± 0.24
Sodium citrate	0.92 ± 2.32	0.96 ± 0.32
Silica	0.55 ± 0.04	0.64 ± 0.11
Malic acid	0.66 ± 0.16	0.61 ± 0.16
Citrus extract	0.45 ± 0.01	0.53 ± 0.22
Olive extract	0.13 ± 0.31	0.17 ± 0.18

Each sample represents a mean of triplicate (n = 3) assays.

**Table 2:** Lethal concentration 50 (LC_50_) at which the natural antimicrobial mixture (AG) reduced the number of *E. tenella* and *E. bovis* oocysts by more than 50%.

Antimicrobial mixture (%)	LC_50_ (%)	
	*E. tenella*	*E. bovis*
0	–	–
0.3	28.7	24.3
0.5	47.9	50.2
1	65.7	63.1
2	81.6	87.3
4	97.3	98.1
10	ND	ND

Each sample represents a mean of triplicate (n = 3) assays.

*ND* not determined.

Based on the in vitro results we utilized the 0.5% concentration of the natural antimicrobial mixture (AG) to determine inhibition of *E. tenella* (Fig. 1A,B) and *E. bovis* (Fig. 1C,D) to infect MDBK and CLEC-213 cells. Following treatment with 0.5% AG of cells only, of parasite only, or treatment of both, the post-infection counts showed significant decreases in the invasion levels among all the treatments and for both *Eimeria* species (*E. tenella*—Fig. 1A,B; *E. bovis*, Fig. 1C,D). AG treatment for 12 h reduced parasite sporulation by 38.6% and 25.2% in *E. tenella* and *E. bovis* oocysts, respectively (Fig. 1E). Sporulation rates after 24 h of 0.5% AG treatment were lower compared to controls, 52.3% and 56.1% for *E. tenella* and *E. bovis*, respectively. After 48 h, 0.5% AG treatment reduced sporulation by 71.2% for *E. tenella* and 76% for *E. bovis*.

### The in vitro effect of robenidine (ROB) by comparison with the antimicrobial mixture on the invasion of *E. tenella* and *E. bovis*

Efficacy of the anticoccidial drug robenidine (ROB) was compared with the antimicrobial mixture against the ability of *E. tenella* and *E. bovis* to invade MDBK (Fig. 1H) and CLEC-213 cells (Fig. 1I). Our results indicated that 0.5% AG was equivalent to the commercial coccidiostat ROB at reducing infection by *E. tenella* and *E. bovis* in MDBK and CLEC-213 cells at 12, 24 and at 48 h post-infection. Inhibition varied between 50 and 82%. Pre-incubation with 5 μg/ml ROB or 0.5% AG had a similar effect on *Eimeria* invasive ability, indicating that in vivo studies were warranted *investigate* its potential anticoccidial effect.

### In vivo inhibition of *E. tenella* infection in chicken broilers

**Figure 2:**
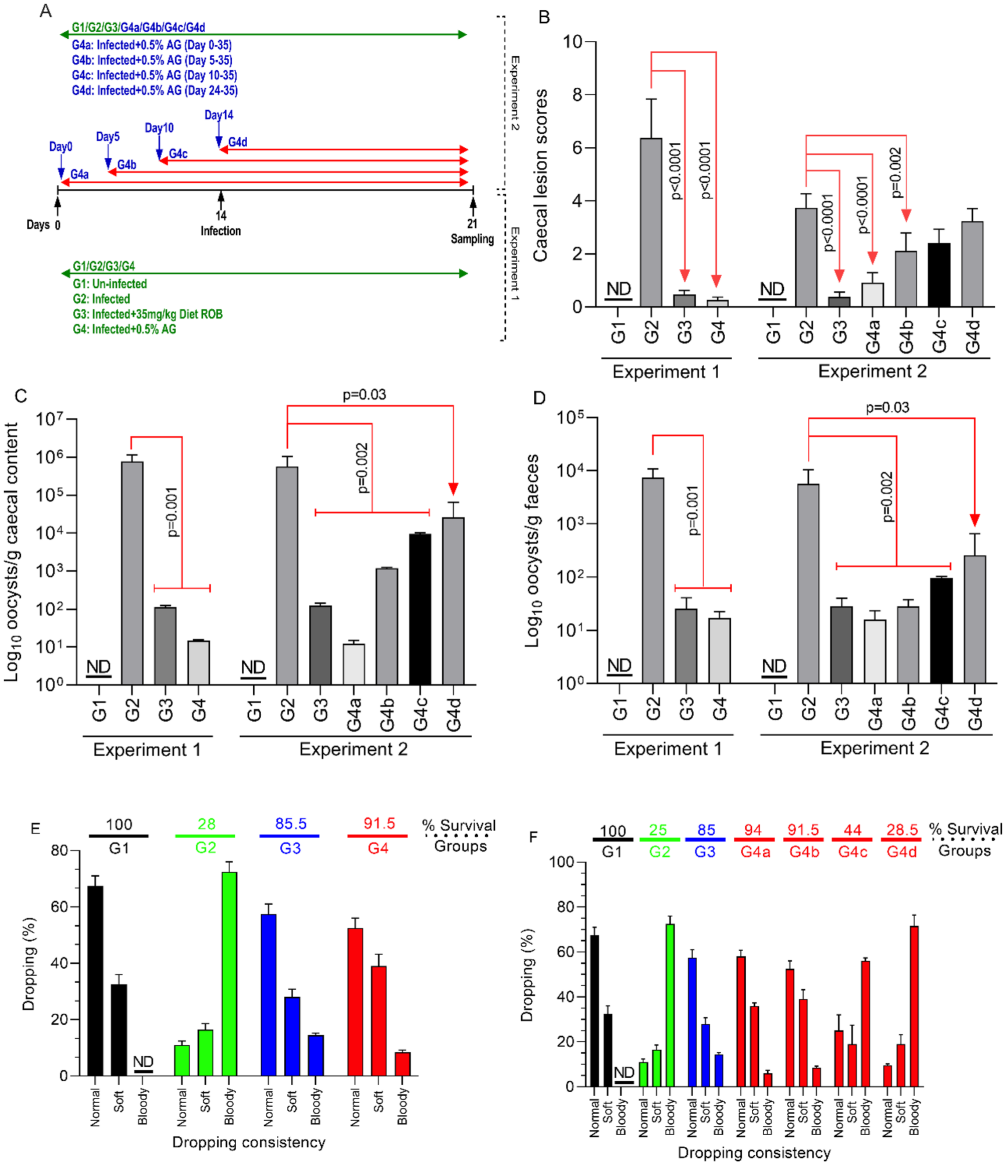
Effect of a mixture of natural antimicrobials (AG) on the in vivo* E. tenella* virulence. (**A**) The in vivo experimental design for Experiment 1 and Experiment 2; (**B**) the caecal lesions scores as recorded during Experiment 1 and 2 and after exposure to 0.5% AG or 35 mg/kg RB; in (**C**) the number of oocysts/g caecal content at day 21 post-infection is presented as Log_10_ followed by the number of oocysts/g faeces at day 21 post-infection in (**D**); the dropping consistency and broiler survival rates during Experiment 1 are shown in (**E**) followed by Experiment 2 in (**F**). All measurements were performed in triplicate and p values are indicated on the graphs indicating significance. *ND* not detected.

**Figure 3:**
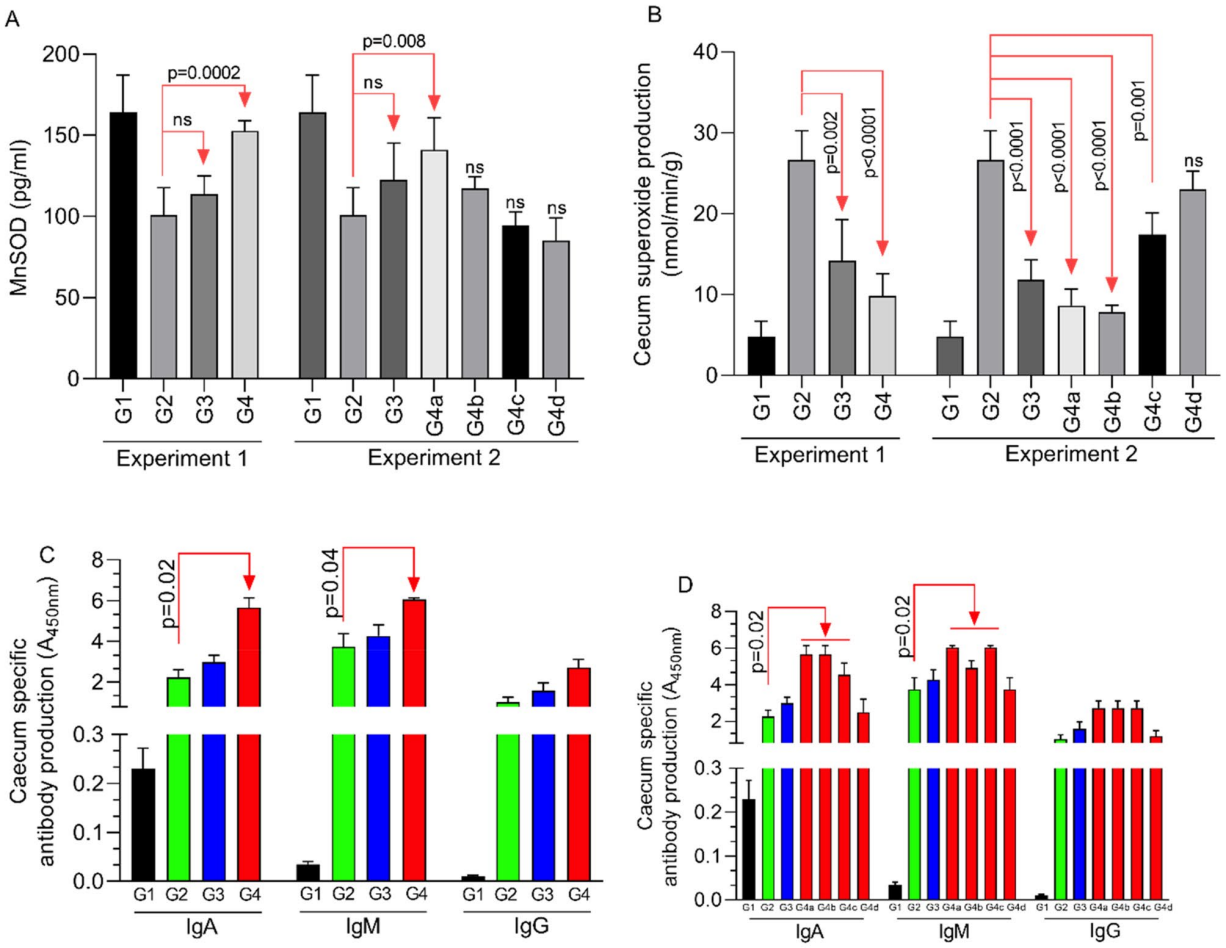
The in vivo impact of the antimicrobial mixture (AG) on MnSOD, superoxide and antibody production levels in the caecal tissue. In (**A**) the levels of MnSOD are shown with the levels of ROS detected in the caecal tissue in (**B**). (**C**) The levels of IgA, IgM and IgG during Experiment 1 followed by the levels of IgA, IgM and IgG in Experiment 2 shown in (**D**). All measurements were performed in triplicate and p values are indicated on the graphs indicating significance.

In vivo inhibition of the natural antimicrobial of *E. tenella* colonisation (Fig. 2A). Gross lesion scores were decreased at 21 d post-infection in the 0.5% AG treated group G4 (p < 0.0001), to a level similar to the ROB-treated infected group G3 (p < 0.0001). Attenuation of caecal lesions was only observed when group G4 received 0.5% AG no later than day 5 post hatch (Fig. 2B). The decrease in lesion score was associated with lower oocyst numbers in the caecal contents (Fig. 2C) and the faeces (Fig. 2D) (p = 0.001). Results suggest (Fig. 3C,D) that 0.5% AG could be included prophylactically in the broilers drinking water, for up to 10 days post hatch, and reduced oocysts in caecal contents (p = 0.002) and in the faeces (p = 0.002). Administration of 0.5% AG at the same time as infection, on day 14 post-hatch, reduced oocyst detection in caecal contents and faeces (p = 0.03), supporting potential prophylactic application. Monitoring the consistency of faecal droppings revealed that in the infected and un-treated group G2, over 75% of broilers had blood detected in the faeces and rate of survival of only 28% (Fig. 2E). However, treatment with 0.5% AG, led to an increase in survival up to 91.5% and less than 15% blood presenting faeces detected in group G4, compared to group G3 where the survival rate decreased to 85% (Fig. 2E). In experiment 2, AG reduced the presence of blood in faeces only if the antimicrobial was applied no later than 5 days post hatch (Fig. 2F). Production performance parameters indicated (Supplementary table 2) that infected and un-treated broilers (G2) had the lowest body weight (590 g) compared to group G4 (784 g). Collectively, results suggest that application of 0.5% AG in the drinking water on d0 or 5 post hatch improved the broiler survival rate via a significant reduction in *E. tenella* infection.

### In vivo reduction of oxidative stress and increase in the immune response in AG exposed broilers

*Eimeria tenella* antigen-binding specific antibodies in the caecal epithelium 21 days post-infection, were higher when treated with 0.5% Ag (group G4), with IgA (p = 0.02) and IgM (p = 0.04) increased compared with the infected and un-treated controls in group G2 (Fig. 3C). Prophylactic treatment with 0.5% Ag from hatch (day 0), day 5 or up to day 10 prior to infection caused higher concentrations of both IgA (p = 0.02) and IgM (p = 0.04) in groups G4a, G4b and G4c compared to group G2 (Fig. 3D). Interestingly the inclusion of ROB in the diet did not significantly influence antibody response suggesting a different mode of parasite inhibition. MnSOD production was increased (p = 0.0002) in the treated G4 group when compared to the infected and un-treated group G2 (Fig. 3A), but not when compared with the ROB treated and infected group G3. Group G4 (AG treated) was only increased when the 0.5% AG was administered from hatch until 21 days of age. Increased MnSOD production was translated in decrease oxidative stress in the form of superoxide in the caecal tissue (p < 0.0001) compared to group G2 (Fig. 3B), which was similar to that of ROB addition (p = 0.002) to the basal diet. In group G the significant decrease in caecal ROS was observed if AG was applied beginning on day 0 (p < 0.0001), day 5 (p < 0.0001) or day 10 (p = 0.001) prior to infection (Fig. 3B). Collectively, our in vivo results indicated that AG treatment could improve the immune response and to reduce parasitic oxidative stress in Eimeria infected chickens.

### The impact of the antimicrobial mixture on the short chain fatty acid (SCFA) production

Inclusion of the antimicrobial mixture in the drinking water of experimental broilers led to significant differences (p ≤ 0.05) between *E. tenella* only challenged and *E. tenella* groups treated with AG or ROB for caecal digesta pH, formic, acetic, propionic acid, n-Butyric and total SCFA concentrations (Table 3).

**Table 3:** In vivo caeca pH and short chain fatty acids levels (SCFA).

Groups	pH	SCFA (µmol/g of caeca digesta)						
		Lactic	Formic	Acetic	Propionic	Iso-butyric	n-Butyric	Total SCFA^c^
G1	6.4	35.7	6.7^a,b^	69.2^b^	31.5^a^	14.1	43.1^a^	206.7^a^
G2	6.1^b^	36.2	7.1^b^	74.3^a,b^	38.4^b^	14.5	46.8^b^	223.4^b^
G3	6.7^a^	34.3	7.8^a^	71.5^a^	30.9^a^	13.9	41.6^a^	206.7^a^
G4	6.8^a^	34.1	7.9^a^	70.9^a^	30.4^a^	14.1	42.1^a^	206.3^a^
SEM	0.12	1.45	1.01	2.33	4.24	0.44	1.15	10.74

Mean values assigned different letter superscripts within a criterion differ, p ≤ 0.05.

^c^Summation of lactic, formic, acetic, propionic, iso-butyric, and n-butyric acids.

Inclusion of ROB in the feed or 0.5% AG in the drinking water led to significant differences in their caecal pH between groups G4 vs. G2, G3 vs. G2. Similar results were observed for the formic acid levels where the inclusion of AG or ROB led to a decrease from 7.9 µmol/g of caeca to 7.1 µmol/g of caeca (G4 vs. G2) and from 7.8 µmol/g of caeca to 7.1 µmol/g of caeca when group G3 was compared to group G2. The acetic acid levels were significantly increased in the infected and ROB treated group G3 when compared to the infected and un-treated group G2 (71.5 vs. 74.3 µmol/g caeca) and between the AG treated group G4 and group G2 (70.9 vs. 74.3 µmol/g ceca). The levels of acetic acid were also significant when all groups were compared to the un-infected and un-treated group G1 suggesting an effect of *E. tenella* infection on the detected levels. Propionic acid measurements indicate that infection has indeed an effect on the detected levels (G2) however, the impact of AG (G4) or ROB (G3) treatment, even though significant, was not different to the control group G1. By analysing the total SCFA detected between groups we can conclude that *E. tenella* challenge increases the levels of SCFA produced (G2) which are significantly reduced when either AG or ROB treatment is applied (groups G3 and G4) to levels similar to those detected in the un-infected and un-treated group G1.

## Discussion

In both monogastrics and ruminants infection by parasites of the phylum Apicomplexa, can lead to significant economic losses, and which costs United States producers more than $450 million^23^ and globally cost more than 3 billion USD^12^. Due to their capacity to exploit the host digestive environment for growth and survival, *E. tenella* can cause infections in poultry and *E. bovis* in cattle^24^, leading to reduced animal growth performance, as well as high morbidity and mortality rates^25^. Mixtures of natural antimicrobials, including organic acids, have been previously shown to inhibit the capacity of parasites (*Cryptosporidium parvum* and *C*. *bovis*) to infect human and bovine epithelial cells in vitro^26^, however, we must understand their activity to be tested in vivo. Our ability to design effective in vivo trials to understand the effects of novel parasitostatic natural antimicrobials has to be based on preliminary results to guide in vitro success when live animals are involved^27^. The aim of this study was to investigate, in vitro, the ability of a mixture of organic acids, to prevent the invasion of MDBK and CLEC-213 cells and subsequently to examine the ability of *E. tenella* to colonise chicken caeca in vivo.

Organic acids, including malic acid mixtures have been shown to reduce the ability of both bacteria and parasites to not only invade intestinal cells, but also to improve immune responses in cultured cells^22,28,29^. Similar combinations reduced bacterial pathogenicity, restored intestinal epithelial integrity, fortified the mucosal barrier^30^, and improved humoral immunity^31^. Present results showed that the natural antimicrobial mixture reduced the amount of *E. tenella* and *E. bovis* DNA detected inside the infected epithelial cells but also the number of viable parasites detected within the cells. Our results further suggest that the antimicrobial mixture impacted not only the host, but also directly inhibited the parasites, reducing their ability to infect avian and mammalian cells.

The unique characteristics of *Eimeria* become evident once sporulation occurs and is able of producing infections at this stage^32,33^. Infection by sporulated *E. maxima* in chickens damaged intestinal villi and reduced nutrient absorption and performance^34^. While some natural antimicrobials were known to be effective against *Eimeria* spp., the impact on sporulated cells remained unknown^35^. The present study demonstrated that mixtures of organic acids reduced the in vitro sporulation of *E. tenella* and *E. bovis*.

Prophylactic treatment against coccidiosis in poultry has been carried out through the inclusion of various drugs (e.g. sulfaquinoxaline, robenidine) in feed^36^. Robenidine (a guanidine derivative) prevents formation of mature schizonts^37^ by inhibiting ATPases and oxidative phosphorylation^38^. In vivo 36 mg robenidine/kg in feed prevented coccidiosis^39^. In the present study, the natural antimicrobial mixture (AG) exhibited a comparable efficiency to robenidine by preventing the infection of MDBK and CLEC-213 cells by *E. tenella* and *E. bovis.*

While many antimicrobials have undergone in vivo testing, there have been few investigations of the underlying biological mechanisms of their mode of action^13,40,41^. Our in vivo study sought to identify the minimum prophylactic duration and gain more information in regard to the biological mechanisms underlying AG treatment. A reduction in oocyst presence in the caecal contents and in faeces was associated with a decrease in intestinal wall lesions and increased the broiler survival rates from 28 to 91.5%. Our results show that an early inclusion of AG in broiler drinking water will be most impactful in reducing coccidial infection. Moreover, from a mechanistic point of view we now know that the antimicrobial mixture (AG) reduced MnSOD production, however this did not occur in Robenidine (ROB) treated birds. Broilers with higher levels of MnSOD had lower tissue superoxide detected, which also was found in the ROB treated chickens. We hypothesized that decreased superoxide in the ROB treated broilers was due to prevention of the formation of mature schizonts and inhibition of the parasite’s respiratory chain, as previously indicated^38^, thereby preventing infection driven superoxide formation.

Cell mediated immune responses to avian coccidiosis involve specific serum antibodies as a protective measure against *E. tenella* infections^42^. In the present study, *E. tenella* infected broilers (G4) treated with 0.5% AG expressed higher levels of IgA and IgM when compared to the infected and un-treated group G2. Other researchers have found similar reductions in IgA, IgG and IgM concentrations when infected broilers were treated with herb polysaccharide extracts^43^; however, in our study no impact was observed on IgG levels. Intestinal immunity is also involved in preventing inflammation^44^ through the biosynthesis of short chain fatty acids (SCFA), well-known for their antagonistic effect against pathogenic bacteria and parasites^45,46^. Our study shows that the total SCFA levels are reduced in the presence of *E. tenella* when either AG or ROB treatment is applied, an observation previously described when infected broilers received a nucleotide-rich yeast extract^45^. At individual level the antimicrobial mixture caused significant differences in formic, acetic, propionic and n-Butyric SCFA production suggesting a possible role in manipulating the microbial populations involved in their biosynthesis. Thus, these results indicate that antimicrobial mixtures can potentially have a significant impact on the immune responses of broilers and could be considered as an efficient intervention at farm level, but that each phytochemical may have a unique mode of action.

## Conclusion

Mixtures of natural antimicrobials have greater efficacy at controlling coccidiosis in animals. But the complex nature of the colonization process requires a tailored combination of in vitro evidence combined in vivo data so the effects of these mixed antimicrobials can be understood and utilized as part of a One Health approach to improving animal health. Previously it has been suggested that combinations of antimicrobials, rather than single compounds, were more efficient in controlling bacterial infections^47^ and our study demonstrates that synergistic effects can be also occur against parasitic infections in vitro and in vivo. Results suggest that mixtures of natural antimicrobials can: (1) modulate the host immune response, (2) reduce parasite-induced host oxidative events, and (3) alleviate clinical signs and growth inhibition associated with coccidiosis. Overall, our results suggest that natural antimicrobial mixtures could have a broad spectrum anti-parasitic effect and had higher efficacy when compared to the well-known anti-coccidial drug Robenidine.

## Material and methods

### Epithelial cell lines, parasites, and antimicrobials

In this study Madin-Darby bovine kidney (MDBK) cells (Sigma-Aldrich, UK) were grown at 37 °C and 5% CO_2_ in DMEM (Gibco, UK) supplemented with 2% foetal bovine serum and 100U/ml penicillin/streptomycin (Thermo-Fisher, UK). The chicken lung epithelial cell line CLEC-213 cells line was grown as previously described^48^. *Eimeria tenella* Wisconsin strain^49^ and *E. bovis* (laboratory own isolate—unpublished), isolated from Holstein Friesian calves, were used to test the antimicrobial effect of AG in vitro. Sporozoite purification was performed following previous methods^50^. The antimicrobial mixture used is known throughout the manuscript as Auraguard (AG) which contained: maltodextrin, sodium chloride, citric acid, sodium citrate, silica, malic acid, citrus extract and olive extract, and the individual antimicrobials were supplied by Bioscience Nutrition, Fedamore, Ireland.

### Determination LC_50_ cytotoxicity and the impact on epithelial cell proliferation (MTT assay)

The methodology used to determine the antimicrobial effectiveness against *Eimeria tenella*, *E. bovis* oocysts was previously described^51^. We have determined the cytotoxicity of individual components (0.05–1 mg/ml) and then combined in mixtures (0–10%). Briefly the antimicrobial mixture was spread on 0.2% Agar and tested in increasing concentrations from 0 to 10% (0; 0.3; 0.5; 1; 2; 4 and 10%). Activity was determined in triplicate in 96 well microplates by incubation for 24 h of an inoculum of 40 µl containing 1.6 × 10^7^ oocysts/ml. The LC_50_ was determined from curves by expressing the number of oocysts according to the antimicrobial mixture concentration by identifying the concentration in which the number of oocysts was equal to half of the initial number. Oocyst counts were performed as previously described^51^. In order to test effects of antimicrobial treatment on epithelial cell proliferation, the cells were cultured as described above and treated with a series of concentrations of AG (0; 0.3; 0.5; 1; 2; 4 and 10%) for 24 h or treated with 0.5% AG for 0, 3, 6, 9, 12, 15 and 24 h to examine the dose or time-dependent AG effect on MDBK and CLEC-213 cells viability was assessed using MTT (3-(4,5-dimethylthiazol-2-yl)-2,5-diphenyl-tetrazolium bromides) assay.

### In vitro infection assay

We performed infections in two types of epithelial cells. Monolayers of MDBK and CLEC-213 cells were prepared in 24-well plates at 0.3 × 10^6^ cells/well. Sporozoites of *E. tenella* and *E. bovis* (0.5 × 10^6^ sporozoites/well) were pre-treated for 30 min at 41 °C−5% CO_2_ with 0.5% AG in DMEM, as previously described^50^. A 0.5% antimicrobial mixture was previously shown to sub-inhibitory in parasite infection assays^26^. Secondly, MDBK and CLEC-213 cells were pre-treated with 0.5% AG for 1 h prior to infection. Finally, a concentration of 0.5% AG was maintained during an infection experiment without pre-treatment of cells or parasites. Sporozoites were added to MDBK or CLEC-213 cells and at 24 h post-infection infected monolayers were washed in phosphate buffered saline (0.5 ml/well). After infection infected cells were washed and fixed with 4% paraformaldehyde and were mounted in DAPI mounding media (Vectashield) (Cole-Parmer, UK). Sporozoites were counted as previously described using a Carl Zeiss inverted microscope^52^. The percentage of infected cells was calculated as mean ± SD of at least four independent replicates.

### The effect of the antimicrobial mixture on *E. tenella* and *E. bovis* oocysts sporulation

The sporulation time was determined by adding 100 oocysts to wells that contained the antimicrobial mixture at a concentration of 0.5% and incubated at ambient temperature 24 °C and oxygen. The suspension was examined by haemocytometer after 12, 24 and 48 h of exposure to determine the percentages of sporulated oocysts. Oocysts sporulated in diclazuril and potassium dichromate (2%) solutions were used as control^53^.

### Anticoccidial drugs versus natural antimicrobial anti *Eimeria* assay

This assay was performed as previously described^54^ with few modifications. Sporozoites (1 × 10^6^) of *E. tenella* and *E. bovis* strains were pre-treated for 1 h at 41 °C, 5% CO_2_ with the anticoccidial compound robenidine (ROB). The anticoccidial compound was used at a concentration of 5 μg/ml in PBS from dimethyl sulfoxide (DMSO). Same protocol was applied for treating the sporozoites with 0.5% mixture of natural antimicrobials (AG). A concentration of 0.05% DMSO and DMEM was used as a control. After incubation, sporozoites treated with either AMP or ROB were resuspended in DMEM and added to MDBK and CLEC-213 monolayers. At 12, 24 and 48 h post infection cells were washed and fixed with 4% paraformaldehyde and were mounted in DAPI mounding media (Vectashield) (Cole-Parmer, UK). Sporozoites were counted using a Carl Zeiss inverted microscope^52^. Infected cell percentage was calculated as mean ± SD of at least four independent replicates.

### In vivo trials

The experimental design was evaluated and approved by the Ethical and Animal Welfare Committee of the Banat University of Agricultural Sciences and Veterinary Medicine, King Michael I of Romania, Timisoara and all methods were performed in accordance with the relevant guidelines and regulations. Survival rate was recorded from day 14 to 21. Faecal samples were collected on at 21 days of age or 7 days of infection. The faecal oocyst numbers being expressed as oocysts per gram of faeces. In Experiment 1, Ross 308 broilers (n = 40), obtained from a local hatchery at 1 d of age, were divided in 4 groups of 10 broilers per group (Fig. 2A). All broilers had ad libitum access to feed and water through the experimental period. Group 1 (uninfected and un-medicated control, G1) and Group 2 (infected un-medicated control—G2) were fed with standard chicken diets from day 0–10 and from day 11–21 (Supplementary Table 1). Chickens in Group 3 (G3) were fed with the daily basal diets containing robenidine (Robenz 66G) (35 mg/kg feed) and Group 4 (G4) received the standard diets and had ad libitum access to feed and water containing 0.5% AG. Experiment 2 was designed to investigate the prophylactic effect of AG and structurally designed similarly to Experiment 1 with the exception that Group 4 was divided in 4 sub-groups (10 broilers each). As described in Fig. 2 Panel A, Group 4a received 0.5% AG from Day 1, Group 4b received 0.5% AG from Day 5, Group 4c received 0.5% AG from Day 10 and Group 4d received 0.5% AG from Day 14 (AG administered through drinking water). All broilers, other than the un-infected control, were challenged with *E. tenella* on day 14 (1 × 10^4^ sporozoites) with the control chickens receiving 2 ml of phosphate buffered saline (PBS). The survival rate was recorded between days 14 and 21. The Gut pathology, stool, and/or sick bird appearance were observed daily unless indicated otherwise in each group. All chickens were sacrificed at 21 days and faecal samples were collected of age. This study was carried out in compliance with the ARRIVE guidelines on animal research. Post-mortem, caecal tissue was assessed for lesions and scored as follows: lesions were scored from 0 to 6: 0 (no lesions), 1 (mild lesions), 2 (moderate lesions), 3 (severe lesions), 4–6 (very severe lesions).

### Superoxide manganese dismutase (MnSOD) and ROS determination in the caecal tissue

Quantification of MnSOD concentrations in the caecal tissue was performed as previously described^55^. Briefly, the tissues were rinsed in ice-cold PBS (0.02 mol/l, pH 7.0–7.2) to remove excess blood, minced the tissues to small pieces and homogenized them in a certain amount of PBS and stored at − 80 °C until further use. A standard curve was prepared for each experiment. Each measurement was performed in triplicate. Superoxide was measured as previously described^56^.

### Measurement of caecal antibodies

To measure the levels of IgA, IgM and IgG we have used a previously described protocol^57^. Briefly, the small pieces of the intestinal tissue were washed with HBSS (with penicillin–streptomycin), and resuspended in 5 ml RPMI-1640 Dutch modified medium containing 100 μg/ml of gentamicin, 40 mM HEPES buffer (pH 7.2), and 2 mM l-glutamine. Following incubation and centrifugation the specific IgA, IgM, and IgG isotypes in all aliquots were determined by ELISA. Immunoglobulin levels were measure by ELISA using the Enzyme-Linked Immunosorbent Assay (ELISA) kit (ab157691 ABCAM) according to the manufacturer’s instructions.

### SCFA determinations

The SCFA were analysed by gas chromatography as previously described^55^. Briefly, 1 g of ceca was mixed 1 ml of H_2_O and 1 ml of 20 mmol/l pivalic acid solution as an internal standard. The solution was mixed and 1 ml of HClO_4_ (perchloric acid) was added in order to extract SCFA by shaking by vortexing for 5 min. The HClO_4_ acid was precipitated by adding 50 ml of 4 mol KOH into 500 ml of supernatant. The addition of saturated oxalic acid, at 40C for 60 min, and centrifugation at 18,000*g* for 10 min. Samples were analysed by gas chromatography using SCION-456-GC with a flame ionization detector.

### Statistical analysis

Statistical analyses were performed using GraphPad software version 9 (https://www.graphpad.com). Data were represented as mean ± SD. Significance was assigned at p values < 0.05 following estimations using the Student *t*.

## Supplementary Information

Supplementary Information.
