# Supplementary material for: The in vitro and in vivo anti-virulent effect of organic acid mixtures against Eimeria tenella and Eimeria bovis
Source: Sci Rep. 2021 Aug 10;11:16202. doi: 10.1038/s41598-021-95459-9 (PMC8355357; doi:10.1038/s41598-021-95459-9)
Supplement: Supplementary file 1 — Supplementary Information. [file 41598_2021_95459_MOESM1_ESM.docx]

**The *in vitro* and *in vivo* anti-virulent effect of organic acid mixtures against *Eimeria tenella* and *Eimeria bovis***

Igori Balta^1,2,3*^, Adela Marcu^3*^, Mark Linton^1^, Carmel Kelly^1^, Lavinia Stef^3^, Ioan Pet^3^, Patrick Ward^4^, Gratiela Gradisteanu Pircalabioru^5^, Carmen Chifiriuc^5^, Ozan Gundogdu^6^, Todd Callaway^7^, Nicolae Corcionivoschi^1,2,3*^

^1^Bacteriology Branch, Veterinary Sciences Division, Agri-Food and Biosciences Institute, Belfast, Northern Ireland, United Kingdom

^2^Faculty of Animal Science and Biotechnologies, University of Agricultural Sciences and Veterinary Medicine, Cluj-Napoca 400372, Romania

^3^Faculty of Bioengineering of Animal Resources, Banat University of Animal Sciences and Veterinary Medicine - King Michael I of Romania, Timisoara, Romania

^4^Auranta, Nova UCD, Belfield, Dublin 4

^5^Research Institute of University of Bucharest, 300645, Bucharest, Romania

^6^Department of Infection Biology, Faculty of Infectious and Tropical Diseases, London School of Hygiene and Tropical Medicine, London, United Kingdom

^7^Department of Animal and Dairy Science, University of Georgia, Athens, GA, United States

**Email addresses:**

Igor Balta: igori.balta@gmail.com

Adela Marcu: adelamarcu@usab-tm.ro

Mark Linton: Mark.linton@afbini.gov.uk

Carmel Kelly: Carmel.kelly@afbini.gov.uk

Lavinia Stef: lavi_stef@animalsci-tm.ro

Ioan Pet: ioanpet@eurofins.com

Patrick Ward: pat@auranta.ie

Gratiela Gradisteanu Pircalabioru: gratiela87@gmail.com

Mariana Carmen Chifiriuc: carmen.chifiriuc@gmail.com

Ozan Gundogdu: ozan.gundogdu@lshtm.ac.uk

Todd Callaway: todd.callaway@uga.edu

Nicolae Corcionivoschi: nicolae.corcionivoschi@afbini.gov.uk

***** Correspondence: [nicolae.corcionivoschi@afbini.gov.uk](mailto:nicolae.corcionivoschi@afbini.gov.uk) and todd.callaway@uga.edu

Agri-Food and Biosciences Institute

18a Newforge Lane

Belfast, BT9 5PX

Northern Ireland, UK

00442890255662

[nicolae.corcionivoschi@afbini.gov.uk](mailto:nicolae.corcionivoschi@afbini.gov.uk)

**Supplementary Table 1**

Chemical composition of basal diet

| Item | Starter | Grower |
| --- | --- | --- |
|  | 0–10 days | 11–21 days |
| Wheat | 54.623 | 57.553 |
| Full fat soya | 12.000 | 12.000 |
| Brazilian GM hipro | 25.000 | 21.000 |
| Lime bulk | 0.717 | 0.700 |
| DCP bulk (18.1% p) | 1.654 | 2.000 |
| Salt bulk | 0.200 | 0.200 |
| Sod.bi-carbonate | 0.199 | 0.166 |
| DL methionine | 0.487 | 0.435 |
| L-lysine | 0.373 | 0.318 |
| Threonine | 0.247 | 0.128 |
| Vitamin+mineral premix | 0.500 | 0.500 |
| Soyabean oil | 4.000 | 5.000 |
| ME Kcal/kg | 2999 | 3081 |
| CP | 23.12 | 21.53 |
| Lys | 1.45 | 1.308 |
| Met+Cys | 1.089 | 0.996 |
| Ca | 0.97 | 0.906 |
| AvP | 0.49 | 0.41 |

**Supplementary Table 2**

Broiler performance

| Age | Index | Experimental group | | | |
| --- | --- | --- | --- | --- | --- |
|  |  | G1 | G2 | G3 | G4 |
| 21 Days | BW | 735 | 590 | 681 | 784 |
|  | FI | 1004 | 932 | 981 | 981 |
|  | FCR | 1.36 | 1.57 | 1.44 | 1.25 |
| *P* values | |  |  |  |  |

BW – body weight

*P*_G2 vs G4_=0.02

*P*_G3 vs G4_=0.04

FI – feed intake

*P*_G2 vs G4_=0.02

*P*_G3 vs G4_=0.04

FCR – feed conversion rates (FI/BW**)**

*P*_G2 vs G4_=0.02

*P*_G3 vs G4_=0.04
